# Indoor Abnormal Behavior Detection for the Elderly: A Review

**DOI:** 10.3390/s25113313

**Published:** 2025-05-24

**Authors:** Tianxiao Gu, Min Tang

**Affiliations:** School of Information Science and Technology, Nantong University, Nantong 226019, China; 2430310044@stmail.ntu.edu.cn

**Keywords:** human activity recognition, fall detection, abnormal detection, sensors, video

## Abstract

Due to the increased age of the global population, the proportion of the elderly population continues to rise. The safety of the elderly living alone is becoming an increasingly prominent area of concern. They often miss timely treatment due to undetected falls or illnesses, which pose risks to their lives. In order to address this challenge, the technology of indoor abnormal behavior detection has become a research hotspot. This paper systematically reviews detection methods based on sensors, video, infrared, WIFI, radar, depth, and multimodal fusion. It analyzes the technical principles, advantages, and limitations of various methods. This paper further explores the characteristics of relevant datasets and their applicable scenarios and summarizes the challenges facing current research, including multimodal data scarcity, risk of privacy leakage, insufficient adaptability of complex environments, and human adoption of wearable devices. Finally, this paper proposes future research directions, such as combining generative models, federated learning to protect privacy, multi-sensor fusion for robustness, and abnormal behavior detection on the Internet of Things environment. This paper aims to provide a systematic reference for academic research and practical application in the field of indoor abnormal behavior detection.

## 1. Introduction

With the development of technology and improvement in living standards, the average life expectancy of people has extended, and the proportion of the elderly population has continued to increase. Population aging has become a global issue. The elderly population is expected to grow from 9.3% in 2020 to 16.0% in 2050 [1].

Safety concerns regarding independent living in the elderly have become increasingly prominent, among which falling has become a notable risk to health. The World Health Organization estimated that 684,000 fatal falls occur annually, making it the second leading cause of unintentional injury-related death after road traffic injuries. More than 80% of fall-related deaths occurred in low- and middle-income countries, with the Western Pacific region and Southeast Asia region accounting for 60%. Falls are the leading cause of injury, death, and disability among adults aged 65 years and older [2], with approximately 10–15% of falls in older adults resulting in a fracture [3,4]. The global age-standardized incidence of falls was 2238 per 100,000 in 2017 [5]. According to the United States Centers for Disease Control and Prevention (US CDC), in 2018, 13.68 million (27.5%) elderly people experienced at least one fall in the previous year [6]. In 2020, 14 million (27.6%) of older adults reported having experienced at least one fall in the previous year [7].

In recent years, sensor technology has developed rapidly, and wearable sensors have become very popular in many fields such as healthcare, entertainment, safety, and commerce [8]. They can provide accurate and reliable information about people’s activities and behaviors, ensuring a safe and reliable living environment. The method based on wearable sensors carries out behavior analysis by collecting human movement data. However, there are many obstacles to its application due to skin–sensor modulus mismatch, leading to poor biocompatibility [9]; unstable voltage output from self-powered sensors [10,11]; motion artifacts contributing to interface noise [12]; and signal distortion caused by material plasticity and cyclic degradation of nanomaterials [13]. Its practical application is often limited by low wearing compliance, insufficient battery life, sensor noise interference, and privacy concerns, especially in long-term monitoring scenarios. With the rapid development of computer vision technology, deep learning algorithms, and edge computing capabilities, video-based anomaly detection methods are gradually emerging. This kind of method relies on the camera or depth sensor, through the non-contact acquisition of human posture, movement trajectory, and scene context information, combined with convolutional neural networks (CNNs), a spatiotemporal graph convolution network [14], and target detection technology, and performs the high precision identification of complex indoor activities and real-time detection of falling events. At the same time, other non-intrusive sensing technologies are also evolving, such as infrared [15], radar [16], WiFi [17,18,19,20], RFID [21], and depth images [22].

Abnormal human behaviors in the room mainly comprise fall behavior. The effective and accurate identification of fall behavior and an immediate distress alarm is crucial to save the lives of the elderly living alone [23,24]. This paper reviews the existing achievements in the field of indoor abnormal behavior detection, integrates scattered knowledge, and critically evaluates the research quality, providing a decision-making basis for theory and practice, and promoting interdisciplinary integration and academic paradigm innovation. Therefore, we retrieved articles on Web of Science based on the keywords “Human Activity Recognition”, “Video Abnormal Detection”, and “Fall Detection” and screened out the contents related to indoor scenes. In Section 2, we present related reviews on abnormal behavior detection. In Section 3, we divide the existing single modality methods into three main categories: sensor-based methods, video-based methods, and other modality methods. In Section 4, we introduce multimodal fusion methods for abnormal behavior detection. We then list the relevant datasets. Finally, the shortcomings of existing techniques and methods are analyzed and suggested for future research methods. The main contributions of this paper are as follows:

(1) We analyze the existing methods and technologies from the perspective of data sources, divide the existing methods into sensor-based, video-based, other modality methods (WiFi, radar, infrared, etc.), and multimodal fusion methods, and analyze the advantages and disadvantages of the existing methods.
(2) We present the challenges and existing solutions for the detection of indoor behavioral anomalies and give suggestions for the development of the field based on the latest innovative content.
(3) According to the latest technology, we combine audio, pressure sensor, robot, and other technologies to build an indoor Internet of Things abnormal behavior detection system, which is expected to provide a more comprehensive security guarantee for the elderly.

## 2. Survey on Existing Reviews

In this section, we searched on the Web of Science platform based on the keywords “Human Activity Recognition”, “Video Abnormal Detection”, and “Fall Detection” and screened out review articles related to indoor spaces. We found that these literature reviews focused on methods, modes, and applications.

### 2.1. Human Activity Recognition (HAR)

HAR is an important area of research in ubiquitous computing, human behavior analysis, and human–computer interaction [25]. With the continuous progress of Internet of Things (IoT) technology, all kinds of smart devices such as smartphones and wearable devices are widely popularized. The built-in sensors in these devices collect rich multivariate time series data, providing a substantial database for human activity identification. For example, sensors such as accelerometers and gyroscopes can accurately record the acceleration, angle, and other information of human movements, making it possible to monitor and analyze human activities. Recent improvements in video pixel resolution have made video-based HAR technology a hot trend. At present, HAR has been widely used in various practical applications, such as public safety [26], human–computer interaction [27], sports [28], healthcare [29], and other fields.

Mukhopadhyay [30] introduced the application of wearable sensors in the medical, entertainment, safety, and commercial fields, with particular emphasis on the importance of monitoring physiological activities in the medical field, but discussed the implementation of the algorithm in a superficial way. Lentza et al. [31] provided a detailed introduction to the latest machine learning-based methods for human activity recognition and abnormal behavior detection. They also introduced challenges faced by HAR and abnormal behavior detection field, including cross-subject recognition [32], transition activity recognition [33], lack of datasets, energy consumption issues, laboratory condition evaluations, and the absence of a general evaluation framework. Zhang et al. [34] discussed in detail the application of deep learning in HAR based on wearable sensors, including the current state of different deep learning algorithms in HAR.

### 2.2. Video Abnormal Detection

Anomaly detection refers to the problem of finding patterns in data that do not conform to expected behaviors [35]. Usually, we adhere to the following definition: video anomalies can be thought of as the occurrence of unusual appearance or motion attributes, or the occurrence of usual appearance or motion attributes in unusual locations or times [36]. Abnormalities are divided into short-term abnormalities and long-term abnormalities [37]. For example, a pedestrian experiencing a sudden fall can be defined as a short-term abnormality, while sitting is generally considered normal behavior. However, if sedentary and stationary for a long time, it should be defined as an abnormal state, because a long period of rest may pose a risk of diseases such as depression [38,39]. Smart video surveillance is of increasing importance, especially in public places such as airports, railway stations, and shopping centers, as well as in smart medical facilities, such as daily activity monitoring and fall detection among the elderly [40]. Popoola and Wang [41] reviewed video-based identification methods and techniques for anomalous human behavior, especially abnormal behavior detection in video surveillance applications.

### 2.3. Fall Detection

Human falls are one of the most critical health issues, especially for elderly and disabled people living alone. Of those who fall, many suffer serious injuries, such as hip fractures and head traumas, which reduce their mobility and independence, and lead to an increased risk of early death [42]. The elderly population is increasing steadily worldwide. Therefore, human fall detection is becoming an effective technique for assistive living for those people [43].

Casilari-Pérez et al. [44] focused on wearable fall detection systems that use artificial neural networks as the core detection algorithm, and explored the feasibility, advantages, and existing problems of these systems in practical applications. Alam et al. [43] discussed in detail the non-invasive (vision-based) fall detection technology based on deep learning. Ehsan and Snidaro [45] presented a comprehensive review of fall detection systems, emphasizing the use of cutting-edge technologies such as deep learning, sensor fusion, and machine learning. The research explored a variety of methodologies and strategies employed in fall detection systems, including the integration of wearable sensors, smartphones, and cameras.

## 3. Single Modality Approach

Sensor modalities are good at high-precision physical measurement and have sufficient environmental robustness, but they are invasive and limited due to their data dimensions. The advantage of a video-based mode lies in providing rich visual information and non-contact collection, but it is vulnerable to environmental interference and has privacy risks [41]. In this section, we analyze existing articles from the single-modal data source perspective, including RGB, skeleton, depth, infrared, acceleration, radar, WiFi, etc.

### 3.1. Sensor-Based Approach

**Figure 1:**
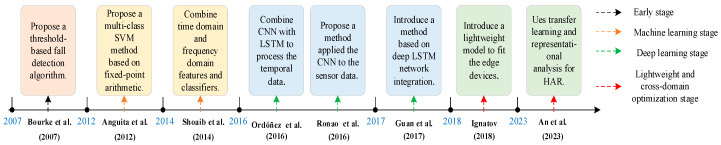
The development process based on the wearable sensor approach [47,48,49,51,52,53,57,61].

We divide the development process of the wearable sensor-based approach into four main stages. The first stage mainly adopts the threshold-based method, the second stage adopts the machine learning method, the third stage adopts the deep learning method, and the fourth stage considers the model lightweight and cross-domain optimization problems. The development process is shown in Figure 1, and we list some classical methods on a timeline.

In the early stages, the threshold method or simple classifier is used based on an accelerometer, gyroscope, etc. Bao and Intille [46] used multi-sensor data to identify daily activities, laying the basis for accelerometer-based HAR. Bourke et al. [47] proposed a threshold-based triaxial accelerometer fall detection method, through the triaxial accelerometer in the trunk and thigh, simulated the fall and daily activities of the elderly acceleration data, and calculated and set the acceleration signal and threshold, using the trunk acceleration signal threshold as a single criterion, to achieve fall detection. This stage method has the advantage of high computational efficiency, but it has insufficient generalization performance in complex activity scenarios.

In the machine learning stage, the time-frequency domain features are manually extracted and combined with traditional machine learning models. Anguita et al. [48] proposed a multi-class hardware-friendly support vector machine method based on fixed-point arithmetic for HAR on a resource-constrained smartphone. By converting the floating-point operation in the traditional support vector machine (SVM) algorithm into a fixed-point operation, this method significantly reduces the computational cost and energy consumption while maintaining a classification accuracy similar to the traditional SVM. Shoaib et al. [49] proposed a physical activity identification method based on built-in motion sensors in smartphones. Combining time domain and frequency domain features and classifiers, they systematically analyzed the performance of each sensor when used alone and in combination. This stage of the study improved the identification accuracy, but the feature extraction process relied on manual design, and the deep information in the temporal data was not fully mined.

With the advancement of deep learning technology, the activity recognition and fall detection of wearable devices have entered a new stage. Deep learning significantly improves recognition accuracy and robustness through automatic feature extraction and end-to-end learning. Plötz et al. [50] proposed a kind of activity identification method based on feature learning, which automatically discovers feature representations suitable for universal computing applications through principal component analysis and deep learning, avoiding the limitations of traditional heuristic feature extraction methods. Ronao and Cho [51] proposed a deep CNN-based method for the HAR of smartphone sensor data to automatically extract features from raw time series data, avoiding the tedious manual feature design in traditional methods. Ordonez and Roggen [52] proposed a deep learning framework called DeepConvLSTM, which combines CNN and Long Short-Term Memory (LSTM), which is suitable for the HAR of multimodal wearable sensors and supports the natural fusion of multimodal sensor data without the need for manual design features. Guan et al. [53] proposed an ensemble learning method based on LSTM networks to train multiple LSTM models by randomly selecting a subset of the training data, which are then integrated for final activity identification. Chen et al. [54] proposed an accelerometer-based fall detection method called ESAEs-OCCCH, combining integrated stacking autoencoders and a convex packet-based classification. Chen et al. [55] proposed a semi-supervised framework for pattern balance to extract and maintain diverse latent patterns of activity, addressing the challenges of insufficient marker data and category imbalance. Clustering (k-means) extracted latent patterns and balanced sampling to avoid changing the data distribution. In addition, they introduced the Recurrent Attention Model, which combines the CNN and LSTM to train the attention mechanism through reinforcement learning and select the most relevant sensor data regions for classification. García et al. [56] proposed a novel data augmentation method to address the issues of insufficient and imbalanced data for fall detection, as well as the limited generalization ability of existing models. By merging the time series of Activities of Daily Living (ADL) and fall events, they generated more realistic training data. The authors employed a combination of CNN and LSTM, where CNN was used to extract spatial features, and LSTM was utilized to handle long-term dependencies in time series.

The current research focuses on achieving real-time user monitoring and cross-domain optimization under low-power-consumption conditions. Ignatov [57] proposed a solution for the user-independent HAR problem that is based on a CNN augmented with statistical features that embrace global properties of the accelerometer time series. It has the benefits of using short recognition intervals of up to 1 s and requiring almost no feature engineering and data preprocessing. Due to the relatively shallow architecture, the proposed algorithm has a short running time and can be efficiently executed on mobile devices in real time. Yhdego et al. [58] used transfer learning methods, combining a pre-trained deep CNN model (AlexNet) to analyze accelerometer sensor data. The acceleration data were transformed into time-frequency images through continuous wavelet transform, and a pre-trained CNN was used to extract general features, solving the problem of small sample training; data augmentation was also performed to enhance the model’s adaptability to changes in sensor positions. Unlike the traditional model, which combined the data from all inertial measurement units (IMUs) into one input vector, the Multi-ResAtt model proposed by Mohammed et al. [59] can process the data of each IMU independently, capture the local features, and then learn the global features through the residual network. The model combines the initial block and the parallel residual module, uses the IMU data, and extracts time series features through a bidirectional gated recurrent neural network and attention mechanism. Tang et al. [60] proposed a new hierarchical segmentation module, which can enhance the representation ability of multi-scale features while maintaining the complexity of the model. Through hierarchical segmentation and feature reuse, the hierarchical segmentation module realizes multi-scale receptive field fusion in a single feature layer. Through cross-group feature splicing, the capture ability of local details and global context information is enhanced to improve the performance without increasing the number of parameters. An et al. [61] proposed a transfer learning and HAR method based on neural network representation analysis. This method retains the general layer parameters of the pre-trained model and only fine-tunes the user-specific fully connected layer, thus significantly improving the recognition accuracy of new users while reducing the training time and energy consumption. Zhou et al. [62] proposed a deep neural network called dfLasso-Net, which was designed with an end-to-end structure for simultaneous sensor selection, feature selection, and classification tasks. Specifically, the authors proposed a two-level weight calculation module, including a sensor weight network and a feature weight network, to measure the importance of sensors and features, and combined the classification network to complete human activity identification.

As shown in Table 1, early threshold approaches offered high computational efficiency but poor generalization in complex scenarios. Machine learning approaches improved accuracy but relied on manual feature engineering and lacked temporal depth. Deep learning approaches enhanced accuracy and robustness via automatic feature extraction and end-to-end learning, though requiring heavy computation and extensive labeled data. Recent advances focus on lightweight real-time models, data augmentation, and transfer learning to address cross-domain adaptability. While sensor-based approaches excel in efficiency, challenges persist in generalization across scenarios, dependency on labeled data, and computational resource demands.

### 3.2. Vision-Based Approach

**Figure 2:**
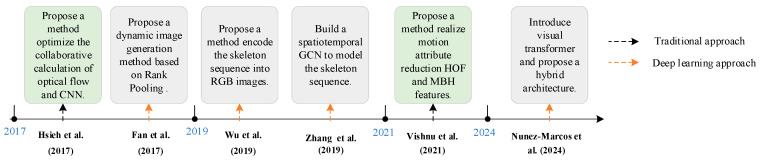
The development process based on the vision-based approach [64,67,69,71,76,82].

The vision-based approach is a better alternative that provides a low-cost solution [63]. Modern artificial intelligence, specifically deep learning, is very effective for this kind of task [43]. In this section, we classify video-based approaches into traditional approaches and deep learning approaches. The development process is shown in Figure 2.

#### 3.2.1. Traditional Approach

Traditional approaches rely on manually designed dynamic features that are computationally transparent but have limited feature expression power. Optical flow can reflect the movement change in pixels between consecutive frames, which is very critical to capture the motion state change in the human body. Hsieh et al. [64] optimized the collaborative calculation of optical flow and CNN to effectively distinguish normal lying position from fall state. Carlier et al. [65] used the custom VGG-16 network to process the optical flow sequence, combined with the time filter to improve the spatial and temporal feature extraction ability. Chhetri et al. [66] innovative fusion of dynamic optical flow and ranking pooling technology to compress the video sequence into dynamic images. Vishnu et al. [67] built a mixed model of fall movement and realized motion attribute reduction through Histogram of Optical Flow (HOF) and Motion Boundary Histogram (MBH) features. Motion History Image (MHI) simplifies subsequent feature extraction and classification tasks by capturing motion information in time series and converting dynamic actions into static image representations. Cai et al. [68] developed a color-coded MHI.

#### 3.2.2. Deep Learning Approach

With the rapid development of deep learning technology, researchers have made significant progress in spatiotemporal information modeling, computational efficiency optimization, and privacy protection through innovative feature characterization methods and network architecture design.

Fan et al. [69] innovatively transformed fall detection into a motion detection problem, and proposed a dynamic image generation method based on Rank Pooling technology. By encoding video clips into single-frame dynamic images and capturing temporal evolution information while retaining spatial features, this method realizes the synergistic application of deep convolutional networks in fall detection and temporal positioning for the first time and provides a scalable framework for sequential action detection.

The method based on human pose modeling detects anomalies by processing human pose-related data, such as the skeleton sequence. Solbach et al. [70] combined stereo vision and CNN pose estimation to achieve the accurate positioning of physical space through three-dimensional coordinate conversion and ground plane detection. Wu et al. [71] encoded the skeleton sequence into RGB images and implemented combined spatiotemporal feature extraction using a lightweight CNN. Zheng et al. [72] built a spatiotemporal GCN to model the skeleton sequence and capture the trunk motion characteristics through the human centerline coordinates. Chen et al. [73] built a three-parameter decision model based on OpenPose key points and constructed a composite criterion through the vertical hip velocity (>0.009 m/s), centerline ground angle (<45°), and aspect ratio (>1). Wu et al. [74] proposed an unsupervised method based on pose estimation, used AlphaPose algorithm to generate a privacy-protected pose graph with the background, and combined it with the prediction error and kinematic features of restrictive generative adversarial network (GAN) to build a fall scoring system, which improved the practicability of the model while reducing the requirements of manual annotation.

In terms of network architecture design, researchers focus on improving the spatiotemporal modeling capability and deployment efficiency. Doulamis [75] developed an adaptive deep network, integrating 3D geometric features and a time delay neural network, to achieve dynamic environment adaptation in the self-calibration framework. Zhang et al. [76] proposed a trajectory-weighted depth convolution descriptor to compress redundant frames by clustering and pooling. Carneiro et al. [77] built a multi-flow VGG-16 network to fuse the complementary features of optical flow, pose estimation, and RGB data. Wu et al. [78] designed a bi-modal deep multi-sample learning framework and innovatively used video-level coarse-grained labels instead of frame-level annotation. By constructing a new objective function to strengthen the feature learning of falling events, and combined with the efficient dual-mode fusion strategy, the data annotation cost is effectively reduced. Kong et al. [79] proposed ETDA-Net to improve the AlexNet structure. Asif et al. [80] designed the privacy protection framework, FallNet. Mobsite et al. [81] built a multi-scale jump-connected segmentation network, through the improved VGG19 encoder and deep separable convolution decoder, combined with the ConvLSTM spatiotemporal analysis module, to further improve the accuracy of fall detection. Nunez-Marcos et al. [82] introduced visual Transformer into this field and proposed a hybrid architecture based on Uniformer, which directly processes raw RGB frames through the synergistic action of convolution and self-attention mechanism. Its sliding window processing mechanism and Adam optimization strategy make the deployment of the model on embedded devices more efficient.

However, deep learning approaches still face several challenges. RGB-based algorithms have insufficient robustness in complex illumination scenarios, and bone sequence methods are limited by pose estimation accuracy, while most networks have limited modeling power for multi-objective interaction scenarios. In Table 2, we summarize the advantages and disadvantages of the video-based approaches. Future research can be conducted in three directions: developing interpretable models with physical constraints to improve decision-making transparency, building a cross-domain adaptive framework to enhance environmental generalization capabilities, and exploring a self-supervised paradigm to mitigate data annotation bottlenecks. These breakthroughs will drive the key leap of fall detection technology from laboratory validation to real-world scenarios.

### 3.3. Other Modality Approach

Although approaches based on wearable sensors and video surveillance occupy a mainstream position in HAR and fall detection [83], their inherent problems of privacy leakage risk, device dependence, and environmental adaptability have always restricted practical applications. In this context, the innovative detection technologies represented by infrared sensing, depth imaging, and wireless signal perception (WiFi/radar) are gradually building the “second front” of indoor abnormal monitoring. The infrared sensor makes use of human thermal radiation features to track the motion trajectory in a lightless environment, which has an inherent advantage in privacy protection. The depth camera overcomes the object through the three-dimensional point cloud reconstruction, and the millimeter wave radar can maintain robust vital signs monitoring in complex scenarios. These technological breakthroughs not only expand the perceptual dimension but also form the possibility of multi-physical field cross-verification, providing a new paradigm for the construction of a universal and highly reliable anomaly detection system.

From the perspective of data sources, we introduce other modality approaches. As shown in Table 3, we list the core technology and targeted problem for each method.

## 4. Multimodal Approach

**Figure 3:**
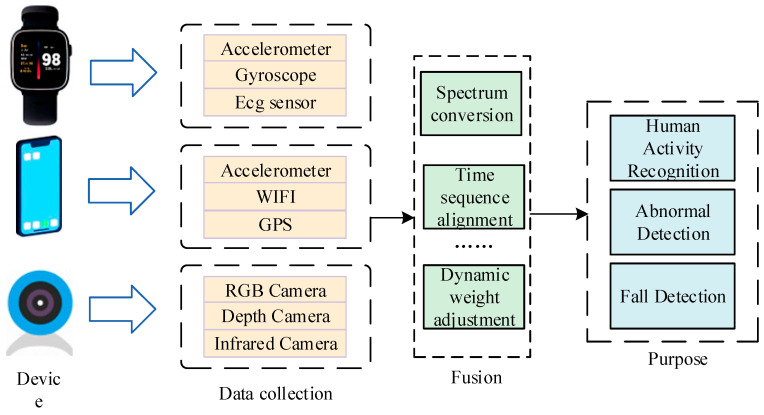
Simple framework for multimodal fusion.

Human activity data captured by a single sensor has limitations. For instance, an accelerometer can only obtain the velocity and acceleration information at a specific position. Relying solely on these data to determine whether a fall has occurred is prone to misjudgment. Therefore, some researchers proposed that multiple sensors be installed in different parts of the human body for detection [84,85,86]. However, the data obtained by such methods are still of a single modality. Now, multimodal data fusion is critical for fall detection systems because it provides more comprehensive information than a single modality. In the early fusion method, the features of different modality sensors are combined by reducing dimensionality and creating new feature vectors. Late fusion or fusion splits the data of each modality sensor, learns the parameters of each modality separately, and combines their probabilistic models [87]. The processing of the multimodal fusion method is shown in Figure 3. After collecting the data from the built-in sensors of each device, the specific identification and detection are realized after a series of modal fusion steps.

Galvão et al. [88] believed that systems based on wearable devices, vision, pressure sensors, and smartphones each have their own advantages and disadvantages, but when used individually, they have issues with robustness. To address this, they proposed a multimodal fall detection system based on deep learning, which combines RGB images and accelerometer data. CNN and LSTM are used to extract spatial features from images and temporal sequence features from accelerometer data. They started with the processing of RGB images with CNNs. Then, they evaluated the processing of accelerometer measures using both CNNs, but with one-dimensional filters, and LSTMs. The CNNs are experts in processing spatial relations, while the LSTMs are useful for processing temporal patterns. Once the individual modalities are processed, they are concatenated and fed to a fully connected neural layer, in a late-fusing strategy. This layer is used as an output of the model and is the basis for the backpropagation training scheme. The proposed model can be deployed in real-world settings using an RGB camera positioned in a room corner and a device equipped with a three-axis (*X*, *Y*, *Z*) accelerometer. A lightweight Linux-based portable device can execute the model, requiring an SD card to store timestamp-synchronized image and sensor data for temporal alignment.

Shu et al. [89] proposed a novel fusion network called ESE-FN (Expansion-Squeeze-Excitation Fusion Network), which aims to effectively fuse multimodal features from RGB videos and skeleton sequences through attention mechanisms at the modality and channel levels. Specifically, ESE-FN firstly implements modal-wise fusion with modal-wise ESE attention (M-ESEA) to aggregate discriminative information in a modal-wise way and then implements channel-wise fusion with channel-wise ESE attention (C-ESEA) to aggregate the multi-channel discriminative information in a channel-wise way. However, this method only integrated RGB and skeletal data, limiting a more comprehensive feature expression.

To address the challenge of effective fusion posed by heterogeneity between different data sources, Qi et al. [90] proposed a novel approach under a federated learning framework that both protects user privacy and utilizes the complementarity of multimodal data. Specifically, the user, as an independent client, does not share local data settings with the server or other clients, thus protecting the security of user information. Each client has a data fusion module that utilizes complementary information from heterogeneous sensors, where time-series data from wearable sensors are converted into images and then fused with visual data from the camera. The local fall detection model was trained based on the local fusion data, and multiple clients jointly trained the global model through Federated Learning (FL). This method improves the identification accuracy of fall detection by input-level data fusion without exposing user data.

A multimodal HAR system for healthcare IoT was developed by Islam et al. [91] Cameras and multiple sensors collect the daily human activities in the form of visual and time series. A deep learning-based fusion network was developed and deployed on the cloud server. This method combines multi-head CNN and a convolutional block attention module (CBAM) to process visual data and uses ConvLSTM to process time-series sensor information.

## 5. Datasets

As shown in Table 4 and Table 5, in this section, we introduce two classes of behavioral detection datasets based on sensors and videos.

### 5.1. Sensor-Based Dataset

The UCI HAR (HAR Using Smartphones) dataset [92] has been collected from 30 subjects performing six different activities. Each person performed six activities wearing a smartphone on their waist. Using its embedded accelerometer and gyroscope, the PAMAP2 physical activity monitoring dataset [93,94] contains data of 18 different physical activities, performed by nine subjects wearing three inertial measurement units and a heart rate monitor. The dataset can be used for activity recognition and intensity estimation while developing and applying algorithms of data processing, segmentation, feature extraction, and classification. The USC-HAD dataset [95] currently includes 14 subjects and 12 daily activities with the sensing hardware attached to the subjects’ front right hip. Each subject completed five trials of each activity in multiple indoor and outdoor scenarios. During the experiment, the sensor is connected to the computer via a flexible cable to record the data, and the experimenter marks the start and end of the activity and the details of the activity on the spot. The dataset also provides MATLAB scripts for visualizing raw data, histograms, and spectrum analysis of accelerometers and gyroscopes. WISDM dataset [96] mainly uses the smartphone’s built-in three-axis accelerometer to record the user’s movement data during daily activities. The goal is to use these sensor data to predict a user’s physical activity through classification algorithms. The dataset consists of 29 volunteers who completed six activities. A total of 4526 samples were collected.

### 5.2. Video-Based Dataset

The URFD dataset [97] contains 70 depth videos collected using a Microsoft Kinect camera at 30 fps (Microsoft, Redmond, WA, USA) that was mounted parallel to the floor. Of these, 30 videos contain a fall, and 40 videos contain various ADL, such as walking, sitting down, crouching down, and lying down in bed. Five people performed two types of falls—from the standing position and from sitting on a chair. The pixels in the depth frames indicate the calibrated depth in the scene. The depth map is provided in a 640 × 480 resolution. The UR dataset contains empty frames. It also contains frames of people entering the scene towards the camera [110]. The SDU dataset [98] contains depth videos collected using a Microsoft Kinect camera. The data that were shared with us contain 1197 depth videos. Of these videos, 997 contain the following ADL: bending, squatting, sitting, lying, and walking. The remaining 200 videos contain a fall, as well as other various ADL. The videos are recorded at 30 fps, with a spatial resolution of 320 × 240, and an average length of 5 s. After applying the sliding window, we obtained 163,573 windows of contiguous frames used for training spatiotemporal autoencoders. The SDU dataset contains empty frames. It also contains frames of people entering the scene from the left and right [110]. The thermal dataset [99] consists of videos captured by a FLIR ONE thermal camera mounted on an Android phone in a room setting with a single view. The videos have a frame rate of either 25 fps or 15 fps, which was obtained by observing the properties of each video. A total of 44 videos were collected, out of which 35 videos contain a fall along with normal ADL, and 9 videos contain only ADL. The spatial resolution of the thermal images is 640 × 480. It also contains frames of people entering the scene from the left and from the right. The Thermal dataset contains 22,116 ADL frames from nine videos [110]. The kinetics dataset [100,101,102,103,104] contains a series of datasets, such as Kinetics-400 [100], Kinetics-600 [101], Kinetics-700 [102], AVA Kinetics [103], and Kinetics 700-2020 [104]. Kinetics-400 contains 400 human action classes, with at least 400 video clips for each action. Each clip lasts around 10 s and is taken from a different YouTube video. The actions are human-focused and cover a broad range of classes, including human–object interactions such as playing instruments, as well as human–human interactions such as shaking hands. The Kinetics-600 and Kinetics-700 datasets are extensions of Kinetics-400. The PKU-MMD dataset [105] is a large-scale multimodal dataset focusing on long continuous sequence action detection and multimodality action analysis. The first phase contains 51 action categories, performed by 66 distinct subjects in three camera views. Each video lasts about 3~4 min and contains approximately 20 action instances. The second phase contains 2000 short video sequences in 49 action categories, performed by 13 subjects in three camera views. Each video lasts about 1~2 min and contains approximately seven action instances [111]. The HMDB51 dataset [106] contains a total of about 6849 video clips distributed in a large set of 51 action categories. Each category contains a minimum of 101 video clips. In addition to the label of the action category, each clip is annotated with an action label, as well as a meta-label describing the property of the clip, such as visible body parts, camera motion, camera viewpoint, number of people involved in the action, and video quality. The NTU RGB+D dataset [107,108] contains 60 action classes and 56,880 video samples. Recently, it has been extended to 120 action classes and another 114,480 video samples in [20]. All the samples were collected from 106 distinct subjects using Kinect sensors. RGB videos, depth map sequences, 3D skeletal data, and infrared (IR) videos are provided for each sample. There is higher variation in environmental conditions compared with previous datasets, including 96 different backgrounds with illumination variations [111]. The Toyota Smarthomes dataset [109] consists of real-world activities of the elderly in their daily lives. The dataset contains 16,115 videos of 31 action classes, and the videos are taken from seven different camera viewpoints.

## 6. Challenges and Future Directions

Indoor abnormal behavior detection faces multiple challenges in practical applications, including the limitations of multimodal datasets, privacy protection issues, adaptability of complex indoor environments, and adaptation of wearable devices. The lack of multimodal datasets limits the ability of the model to understand dynamic behavior, while privacy issues have caused widespread controversy in data collection and use. The lack of robustness in a complex environment affects the reliability of the system, while the adaptation of wearable devices directly affects user experience and data accuracy. Solving these problems requires not only technological innovation but also the combination of practical application scenarios to promote the implementation of technology in the real world.

### 6.1. Multimodal Dataset Issues

Currently, most datasets are limited to a single mode and lack synchronous, multimodal data, making it difficult to capture spatiotemporal features of movements. Most of the existing multimodal datasets are collected in controlled environments, where activities are usually performed by volunteers, different from the real-world situation. Additionally, there is insufficient movement diversity in the dataset, as well as missing samples of different ages, body sizes, and health statuses. Rough granularity, such as “after decline” and “still after decline”, influenced the model’s understanding of the continuous motion. There is also a lack of specific test sets designed for specific scenarios. Therefore, collecting multimodal data from uncontrolled environments to enable large and challenging benchmarks is critical to further facilitate practical applications [112]. Sigurdsson et al. [113] proposed a method called “Hollywood in Homes” to collect real and diverse video data of daily home activities, crowdsourcing participants to record videos at home following scripts, and collect these videos for annotation.

However, collecting large amounts of human movement data is expensive, time-consuming, and challenging. GAN and the diffusion model can be used to generate diverse samples to simulate different ages, body sizes, action speeds, and environmental interferences. Alzantot et al. [114] and Wang et al. [115] used GAN to synthesize the sensor data using existing sensor data. Ramponi et al. [116] designed a conditional GAN-based framework to generate new irregularly sampled time series to increase unbalanced datasets. With the improvement in game engine technology, current games have improved significantly in picture quality and openness, and high-quality, open-world games can provide us with a rich multimodal dataset. Game data not only have a wide variety of scenes and actions but can also simulate a variety of special environments and conditions to make up for the lack of real datasets. In addition, game data also have the advantages of accurate labeling, easy access, and strong scalability. By combining real data with game data, we can build a more comprehensive and rich dataset of human movements to provide a more diverse sample for model training. This can not only help to improve the model’s ability to understand continuous movements and complex scenes but also to further promote the development of human action recognition in practical applications. For example, Sultani et al. [117] proposed a disjoint multi-task learning framework that trains classifiers by alternating real data, game data, and data generated by GANs.

### 6.2. Privacy Issues

The RGB camera records information about the user’s face, clothes, and indoor layout, exposing sensitive information once the data are leaked. However, in the multi-person residence scene, it is sometimes difficult to distinguish different individual activities through video, which requires combining face recognition to further aggravate privacy disputes. The temporal relationship of the data collected by wearable devices can also infer human daily behaviors and habits, which, to some extent, also damages people’s privacy rights and interests. Malekzadeh et al. [118] proposed a deep autoencoder, which solves the problem of the balance between user privacy leakage and data practicability in mobile sensor data. Some researchers adopted the federated learning method to solve the problem of data privacy [119,120,121,122]. However, with the increase in the number of users, especially after reaching a critical value, the data distribution varies greatly, the model aggregation efficiency decreases, and the real-time performance is affected. Furthermore, the federated learning method has certain requirements for GPU resources, which limit its actual deployment in low-power devices (such as mobile phones, mobile watches, etc.). Most of the work was carried out using an RGB input, which is also of concern for subject privacy. To protect the privacy of the subjects, more work can be conducted using thermal and infrared sensors. The depth camera uses an infrared light to capture the subject. These types of cameras can also be used in low-light conditions [43].

### 6.3. Indoor Environmental Issues

Strong backlight or low light blurs body contours in RGB videos, and the traditional algorithm-dependent bone keypoint detection fails. Infrared cameras can alleviate light problems, but they cannot penetrate clothing to detect posture, and high-temperature objects may produce interference signals [123]. In addition, the existing algorithms are mostly based on complete human body detection, and the false detection rate is high when the local limb visibility is poor. In multiple scenes, body crossing and furniture blocking the body when falling often lead to detection failure [124]. Complex interior textures may be misjudged as human movements. Pet activities can also easily trigger false alarms. Existing methods need to be improved to enhance their robustness in complex environments.

To address these challenges, several strategies can be employed. Firstly, integrating advanced image processing techniques such as super-resolution and denoising algorithms can enhance the clarity of RGB videos even in adverse lighting conditions. This can help restore the blurred human contours, making it easier for traditional algorithms like OpenPose to detect skeletal keypoints accurately. Furthermore, combining multiple sensor modalities—such as RGB, thermal, and depth cameras—can provide a more comprehensive view of the scene. While infrared cameras may have limitations in detecting postures through clothing and can be disturbed by high-temperature objects, depth cameras can offer additional spatial information. This fusion of data from different sensors can improve the robustness of the system by compensating for each sensor’s weaknesses.

Moreover, developing algorithms that can work with partially visible bodies is crucial. Instead of relying solely on full-body detection, these algorithms should be able to accurately identify and track limbs even when they are partially occluded by furniture or other individuals. Machine learning models trained on diverse datasets containing various occlusion scenarios can significantly improve the detection accuracy in such cases. To mitigate the issue of indoor textures being misinterpreted as human actions, context-aware algorithms can be implemented. These algorithms should be able to distinguish between real human movements and background patterns, such as carpet designs or TV content. Incorporating semantic segmentation techniques can help in differentiating between humans and other objects in the scene. Additionally, to reduce false alarms triggered by pet activities, the system can be equipped with pet detection capabilities. By training the model to recognize and ignore pets, the system can focus solely on human movements, thereby minimizing false positives.

In conclusion, enhancing the robustness of existing methods in complex environments requires a multifaceted approach, combining advanced image processing, multimodal sensor fusion, partial body detection algorithms, context-aware models, and pet detection capabilities.

### 6.4. Wearable Device Issues

Wearable devices include smart watches, wristbands, chest bands, ankle rings, etc., and the location of different devices may affect data collection. For example, the data collected by wrist devices and chest bands are very different, and position shifts can lead to inconsistent signals [125]. Too-tight device wear may affect comfort, and too-loose wear will cause the device to move, especially during strenuous exercise or falls; the device may also slide, leading to increased sensor data noise. People of different ages and body sizes may have different problems with wearing the same device [126]. The elderly’s skin may be more fragile, and wearing it for a long time may cause discomfort or skin allergies. In addition, obese users may be unable to fit it close into the skin because of their body size, affecting the accuracy of the sensor. For sensor contact problems, fall detection relies on accelerometers and gyroscopes, where the data can be distorted if the device moves [127]. Battery life and maintenance issues are also part of adaptation, and frequent charging can be a burden for the elderly [43]. If the device needs to be charged every day, users may forget or have trouble, leading to interrupted use. Finally, the current wearable devices are mainly smart watches and wristbands, which are slightly more expensive. Currently, wearable devices or mobile devices on the market range in price from tens of dollars, hundreds of dollars, to thousands of dollars [126]. Each family can selectively purchase according to their own actual situation. However, with the gradual increase in the global elderly population, if governments uniformly equip such devices for the elderly across the country, the cost would be high and difficult to achieve. Researchers should minimize the manufacturing cost as much as possible without compromising performance. In addition, adopting video-based detection technology and using sensor devices for assistance is also a good option.

Traditional sensors are mostly based on metal and semiconductor materials, which are insufficiently flexible, portable, biocompatible, inconvenient, and comfortable, so they are difficult to adapt to the high requirements of sensors for the new generation of intelligent sensing devices [9]. Flexible sensors have achieved remarkable breakthroughs in motion detection and health monitoring, with their superior deformability effectively addressing critical issues such as poor skin conformity and wearing displacement [9,128]. Furthermore, innovative designs incorporating self-powered nanomaterials with elastomers have emerged as promising solutions to power supply challenges [129].

Researchers in this field can propose a modular design, allowing users to adjust the wearing position according to their needs; propose an adaptive algorithm to automatically calibrate the position change in equipment; use biocompatible materials to reduce skin irritation; and personalize settings to adjust the sensitivity and threshold according to different users. User education and feedback mechanisms are needed to help users correctly wear and maintain the device, while collecting user feedback to improve the design.

### 6.5. System Integration and Model Deployment Issues

For multi-sensor, multi-device, and multimodal approaches, the problem of “1 + 1 < 1” may arise. Therefore, we need to consider their data fusion methods and the integration methods for actual deployment. Mukhopadhyay [30] believed that the sensor network should be selected based on cost, performance, ease of configuration, addition of extra sensor nodes, range, power consumption, etc. He compared different IEEE communication protocols in five aspects: range, rate, bandwidth, network topology, and application. Bhat et al. [130] developed custom integrated circuits and hardware accelerators that perform the entire HAR pipeline with significantly lower power consumption than mobile or GPU-based platforms.

In addition, most methods detail the models proposed and their performance on the datasets. However, researchers have not fully considered the issues of model deployment. For instance, some methods have achieved decent theoretical data by relying on complex models and efficient GPUs, but the deployment cost in real life is quite high. Islam and Nirjon [131] present an architecture for embedded systems that dynamically schedules DNN inference tasks to improve inference time and accuracy.

### 6.6. Future Directions

We observed that the discussion on audio data is relatively scarce in the field dataset. The potential of audio data for fall detection has not been fully recognized. Currently, the tools and development resources to support audio fall detection are relatively scarce. Recently, Dibble and Bazzocchi [132] proposed a novel multimodal multi-perspective dataset called BIMP, which combines visual and audio data to capture unique environmental fall audio features through multi-perspective recordings, providing a multi-angle basis for sound-based fall detection. At the same time, the dataset has blurred personal information such as faces and tattoos to meet the privacy protection requirements.

We can install audio equipment on the carpet and floor tiles to collect the sound of a fall and judge whether a fall has occurred according to the sound. However, a single audio device is susceptible to interference by ambient noise; therefore, a combined pressure sensor would be an ideal choice. When the audio device captures the fall sound, the reading of the pressure sensor can be used to further confirm whether the fall event has occurred, especially by detecting whether the values match the body weight. In addition, we should consider embedding wearable sensors such as accelerometers into the teeth of the elderly. Given that many elderly people face dental problems, the use of sensor-equipped dentures implanted into the mouth can effectively solve the comfort and forgetting problems of traditional wearable devices.

The penetration rate of intelligent robots for home use is also gradually rising. The RGB camera installed on the robot can realize the collection of multi-perspective video data, thus overcoming the limitation of the single perspective of the traditional fixed camera.

**Figure 4:**
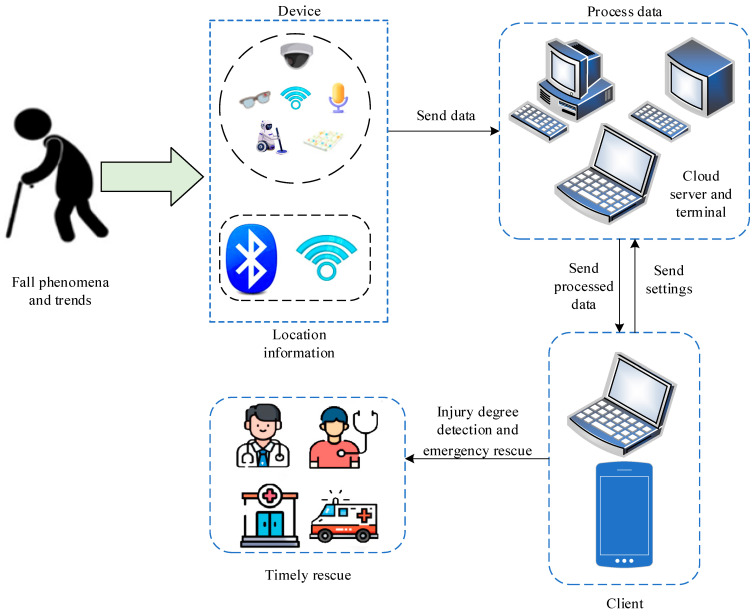
Indoor Internet of Things anomaly detection framework.

All of the above devices can be combined to build an indoor IoT environment. As shown in Figure 4. In this environment, data from a variety of sensors and devices can be transmitted to the central control system in real time. The system uses advanced algorithms to analyze and process these data to realize real-time detection and responses to elderly falls. For example, when an audio device detects a sound suspected of a fall, the system can immediately activate a pressure sensor for detection and synthesize the analysis combined with video data to confirm whether a fall has actually occurred. Meanwhile, the WiFi detection signal immediately locates the elderly person’s position and uploads the location information to the terminal server. In addition, the system can also carry out intelligent early warnings and reminders according to the daily behavior patterns and habits of the elderly. For example, when the system detects that the elderly frequently walk around or do strenuous exercise in a specific period of time, it can timely remind the elderly to pay attention to safety and prevent the occurrence of accidents such as falls. The system can also be connected to the emergency rescue service, so that once a fall is detected, it automatically calls the rescue phone to ensure that the elderly can receive timely assistance.

In summary, the construction of an indoor IoT environment, combined with the data collection and analysis of a variety of sensors and devices, can provide a more comprehensive and accurate solution for the fall detection of the elderly. This can not only improve the quality of life of the elderly but also reduce the pressure on family and society.

## 7. Conclusions

This paper comprehensively reviews the progress of the field of indoor abnormal human behavior detection, with a focus on techniques for fall detection. By combining methods based on sensors, video, infrared, WiFi, radar, depth, and multimodal fusion, the advantages and disadvantages of different technical paths and their applicability in complex scenarios are revealed. This paper suggests that the existing methods still present an imbalance between accuracy and practicality, and the scarcity of multimodal datasets, insufficient privacy protection, and environmental interference restrict the large-scale application of this technology. Future research can focus on the following directions: augmenting diversified datasets with generative models and game engines; enhancing privacy protection through federated learning and deidentification technologies; improving detection robustness in complex environments with multimodal sensors and deep learning; and developing wearable devices with low power consumption and high comfort to optimize user experience.

## Figures and Tables

**Table 1:** The advantages and disadvantages of sensor-based approaches.

Stage of Development	Approaches	Advantages	Disadvantages
Early stage	Simple classifier [46] and threshold [47]	High computing efficiency. Simple implementation, suitable for resource-constrained devices.	Insufficient generalization performance and poor effect in complex scenarios. Relying on manually set thresholds, with poor adaptability.
Machine learning stage	Traditional machine learning model [48] and manual feature extraction [49]	The recognition accuracy is improved compared to the threshold method. The expression ability is enhanced by combining time-frequency domain features.	Feature extraction relies on manual design, which is time-consuming and may miss deep information. Insufficient capture of long-term dependencies on time series.
Deep learning stage	Deep learning models (CNN [50,51], LSTM [53], hybrid models [52,55,56])	Automatically extract features to reduce manual intervention. End-to-end learning to enhance accuracy and robustness. Supports multimodal data fusion.	High demand for computing resources. Reliance on a large amount of labeled data. High model complexity and difficult deployment.
Lightweight and cross-domain optimization stage	Model compression [60], transfer learning [58,61], and attention mechanism [59,62]	High real-time performance, suitable for mobile devices. Strong adaptability across users/devices. Reduces the need for data preprocessing and feature engineering.	Model lightweighting may sacrifice some performance. Transfer learning relies on the data distribution of pre-trained models. Some methods still need to adjust user-specific parameters.

**Table 2:** The advantages and disadvantages of video-based approaches.

Method Type	Advantages	Disadvantages
Traditional approach [64,65,66,67,68]	The calculation is transparent, and the implementation is simple.	The ability to express features is limited.
	Dynamic features that rely on manual design do not require complex models and have lower computational costs.	Poor adaptability: Insufficient robustness to complex scenarios such as illumination changes and multi-object interactions.
Deep learning approach [69,70,71,72,73,74,75,76,77,78,79,80,81,82]	Powerful spatiotemporal modeling capability: Automatic feature extraction.	High computing cost: Complex networks require a large amount of resources and are difficult to deploy in real time.
	Reduce data requirements through self-supervised or weakly supervised learning	Poor interpretability: The decision-making of the black box model lacks transparency.
		Insufficient robustness: The performance of the RGB algorithm declines under complex lighting conditions, and the skeleton method is limited by the accuracy of pose estimation.

**Table 3:** Other modality approaches.

Data Source	Method	Proposer	Core Technology	Targeted Problem
Infrared	AIR-Net	Munsif et al. [15]	EfficientNetB7 + CBAM (Convolutional Block Attention Module) + BiLSTM (Bidirectional Long and Short-Term Memory); Fine-tune InceptionV3 to extract scene context information	Infrared images are blurred, have missing textures and insufficient feature extraction, and inadequately use context information.
Radar	PCC-DT	Wang et al. [16]	The threshold determines the high power density region; Hampel filter denoising	The DT (Doppler time) diagram has many redundant information and large noise interference, which leads to detection errors.
WIFI	WiFall	Wang et al. [17]	CSI (Channel State Information) time-frequency features + weighted moving average noise reduction + SVD (Singular Value Decomposition) dimensionality reduction + SVM / random forest	Detection falls based on WiFi signal and daily activities.
	-	Wang et al. [18]	CSI phase difference ratio + time-frequency domain power steep drop mode	Automatic segmentation and detection of falls during natural continuous activity.
	ABLSTM	Chen et al. [19]	Bidirectional LSTM + attention mechanism-weighted features	Differential in feature contribution of passive activity recognition in WiFi CSI signal.
	FallDar	Yang et al. [20]	Human trunk speed characteristics + VAE (DNN-based Generative Model) generated adversarial data + adversarial learning de-identity information	The influence of environmental diversity, action diversity, and user diversity on WiFi detection.
RFID	TagCare	Jalal et al. [21]	RSS (Received Signal Sntensity) static detection + DFV (Doppler Frequency Values) mutation detection; wavelet denoising + SVM classification	Passive RFID tag detects the status of the elderly living alone and improves the accuracy of fall identification.
Depth	Multi-fusion features of an online HAR system	Zhu et al. [22]	Depth contour + skeletal joint features (trunk distance, joint angle, etc.) + vector quantification + HMM (Hidden Markov Model) online identification	Online activity segmentation and recognition, fusion of space-time multi-features to improve robustness.

**Table 4:** Sensor-based dataset.

Dataset	Device	Activity Category	Subjects	Characteristic
UCI HAR [92]	Smartphone (accelerometer + gyroscope)	6	30	Manual annotation, clear division, basic action recognition support
PAMAP2 [93,94]	IMU+ heart rate monitor	18	9	Supporting activity identification and intensity estimation, containing multimodal data
USC-HAD [95]	MotionNode (accelerometer + gyroscope + magnetometer)	12	14	Support for indoor and outdoor scenes, and provide MATLAB analysis tools
WISDM [96]	Smartphone (accelerometer)	6	29	The goal is to classify daily activities with a moderate amount of data

**Table 5:** Video-based dataset.

Dataset	Modalities	Activity Categories	The Number of Videos	Characteristic
URFD [97]	D	2(Fall+ADL)	70	Contains empty frames and characters in the scene for fall detection.
SDU [98]	D	6(Fall+ADL)	1197	Generates a 163,573-window training model with empty frames and characters in and out of scenes.
Thermal [99]	Thermal imagery	2(Fall+ADL)	44	Thermal imaging data, containing a large number of empty frames and characters entering the scene.
Kinetics [100,101,102,103,104]	RGB	400–700	>10,000	Covers a wide range of human-interactive movements, suitable for complex action recognition.
PKU-MMD [105]	RGB+D+IR+Skeleton	51 (Phase1)/49 (Phase2)	1076	Multi-view, long continuous sequence, supporting action detection and multimodal analysis.
HMDB51 [106]	RGB	51	6849	Challenges to the camera motion, need to align frames, labeled according to action category and scene attributes.
NTU RGB+D [107,108]	RGB+D+IR+Skeleton	60→120	56,880→114,480	High environmental diversity and support for multimodal action recognition.
Toyota Smarthomes [109]	RGB+D+Skeleton	31	16,115	Real family activity scene, including object interaction, multi-perspective coverage.

## Data Availability

The data analyzed during the current study are available from the corresponding author upon reasonable request.
